# Diagnostic accuracy and image quality evaluation of ultrashort echo time MRI in the lungs

**DOI:** 10.1097/MD.0000000000040386

**Published:** 2024-11-08

**Authors:** Funan Wang, Xiaoxia Li, Chong Lin, Liuhong Zhu

**Affiliations:** a Department of Radiology, Xiamen Branch, Zhongshan Hospital, Fudan University, Xiamen, Fujian, China; b Xiamen Municipal Clinical Research Center for Medical Imaging, Xiamen, Fujian, China.

**Keywords:** CT, MRI, pulmonary nodules, ultrashort echo time

## Abstract

This study evaluates the diagnostic accuracy of ultrashort echo time (UTE)-MRI for detecting pulmonary nodules and image quality. A total of 46 patients at our hospital underwent unenhanced computed tomography (CT) and UTE-MRI. The image quality and number of nodules detected using CT were used as the gold standards. Three diagnostic radiologists independently recorded the image quality (visibility and sharpness of normal anatomical structures) of the CT and UTE images and the number of pulmonary nodules detected. The diagnostic accuracy, subjective image quality, and consistency between observations were statistically analyzed. Among 46 patients, 36 (78.2%) had pulmonary nodules on CT images, whereas 10 patients (21.7%) had no pulmonary nodules. A total of 48 lung nodules were detected, 3 of which were ground-glass opacities. UTE-MRI revealed 46 lung nodules. Compared with CT, the sensitivity of all MRI readers for detecting lung lesions was 95.8%, and the 3-observer agreement was nearly perfect (*P* < .001, Kendall W^a^ [Kender Harmonious Coefficient] = 0.913). The overall image quality score of the observers was high, ranging from good to excellent, and the consistency of the subjective UTE-MRI image quality was good (Kendall W^a^ = 0.877, *P* < .001). For tracheal display, the subsegment of the bronchus was displayed, and the wall of the tube was clearly displayed. The difference in the W^a^ values between the observers was 0.804 (*P* < .001), indicating strong consistency. For blood vessels, subsegment blood vessels could also be displayed with clear walls and uniform signals (Kendal W^a^ = 0.823, *P* < .001), indicating strong consistency. Compared to CT, UTE-MRI can detect pulmonary nodules with a high detection rate, relatively good image quality, and strong consistency between observers. The development of UTE-MRI can provide a novel imaging method for the detection and follow-up of pulmonary nodules and diagnosis of pneumonia by reducing ionizing radiation.

## 1. Introduction

Although CT is the preferred method for detecting lung nodules, its slow acquisition, susceptibility to breathing and cardiac movements, and limited soft tissue and water content in the lung tissue contribute to a low signal-to-noise ratio,^[1]^ significantly limiting its use. Lung magnetic resonance imaging has garnered increasing attention for the diagnosis and characterization of lung lesions.^[2,3]^ Recent studies have demonstrated its ability to detect lung lesions and provide more information about their heterogeneity than CT owing to its higher intrinsic soft tissue contrast.^[4]^ However, the role of MRI, including diffusion-weighted imaging, in nodule evaluation remains unclear, with conflicting results.^[5]^

Ultrashort echo time MRI (UTE-MRI) has shown promise for pulmonary applications because it is less affected by rapid T2* attenuation and respiratory motion.^[6–8]^ UTE technology enables the reduction of TE time to the microsecond level (32 μs), making it particularly suitable for imaging organs with low proton content and heterogeneous magnetic fields, such as lung tissue^s^.^[9]^ Ohno et al concluded that UTE-MRI effectively revealed ground-glass opacity, consolidation, and other lung abnormalities, which is consistent with CT findings.^[10]^

To the best of our knowledge, the utility of UTE-MRI sequences for detecting pulmonary nodules and assessing image quality has rarely been studied, and their diagnostic performance, including subjective image quality and interobserver consistency, is not yet clear. Therefore, the objective of this study was to estimate the diagnostic accuracy and subjective image quality of UTE-MRI for the detection of lung nodules, and to analyze interobserver consistency.

## 2. Materials and methods

### 2.1. Inclusion criteria

**Figure 1.**
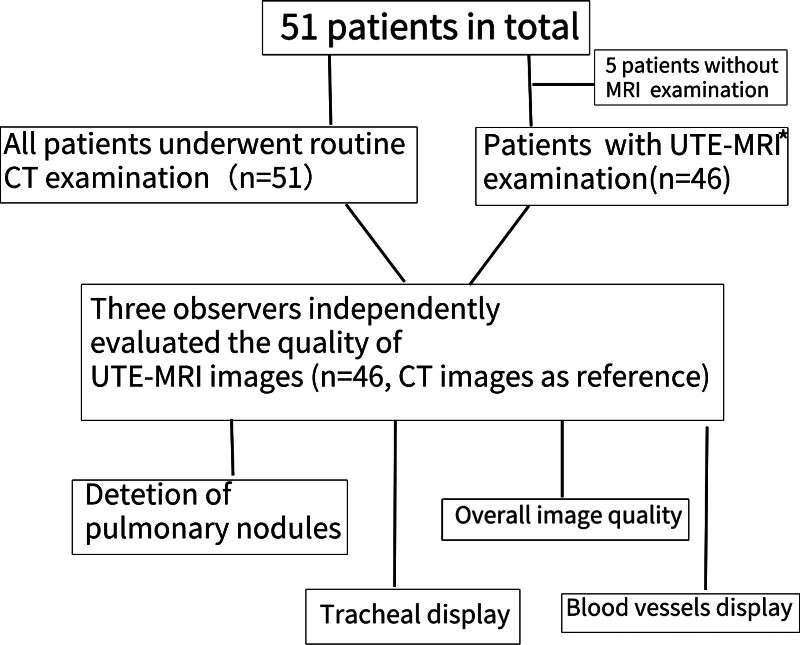
The flow of the study. *UTE-MRI = ultrashort echo time MRI.

The study was reviewed by the ethics committee of our hospital and all patients provided signed informed consent. The workflow of this study is illustrated in Figure 1. Patients who underwent routine CT within 24 hours from November 2018 to October 2021 were selected for UTE-MRI examination. The inclusion criteria were as follows: 1. The patient completed a chest CT scan, and 2. Patients who voluntarily participated in the MRI examinations, and 3. There were no contraindications for MRI examination. Patients with severe pneumonia, acute pulmonary edema, or diffuse emphysema, and those who could not tolerate MR examinations were excluded. Patients under 18 years of age, pregnant women, and others unsuitable for MRI, such as pacemaker implantation and claustrophobia, were also excluded.

### 2.2. CT examination

A 64-slice spiral CT scanner (uCT 760; United Images, China) was used for routine CT. Patients were asked to hold their breath after inhalation. All patients were placed in a supine position with both upper limbs lifted and scanned from the lung tip to the lung floor. The scanning parameters were as follows: collination width, 64 × 0.5 mm; pitch, 1.5; speed, 0.5 seconds; tube voltage, 120 kV; reference tube current, 110 mAs (automatic tube current technology); and image matrix, 512 × 512. Images were reconstructed with a layer thickness and 2.0 mm and a layer spacing of 2.0 mm using a 30% iterative reconstruction technique.

### 2.3. MRI examination

All patients underwent 3T routine magnetic resonance imaging (Discovery 750w, General Healthcare, USA) using a multichannel phased-array body receiving coil. The following parameters were used: supine position, advanced foot, calm regular breathing training, axial, sagittal, and coronal UTE scanning (time of repetition: 10 ms, time of echo: 0.032 ms, 320 × 75, layer thickness 2.0 mm, without interlayer gap, flip angle = 9°, acquisition bandwidth 125 kHz, without fatty suppression technique, and matrix 380 × 210). The sequence was prospectively gated using respiratory data. The gating window was set to 30%. Repetitiontime was maintained at 10.4 ms during gating. The pulse duration of the FPGR is used. A total of 256 readouts were acquired per K-space and undersampling was performed. The temporal distribution of readouts over time was a cone. A total of 32 receiving channels were used. The scanning time was approximately 3 minutes 30 seconds in the coronal and sagittal views, and approximately 7 minutes in the axial view.

### 2.4. Data analysis

Three different observers, who independently analyzed all the datasets, had 12 years (Wang), 4 years (Lin), and 5 years (Li) of experience in thoracic radiology. The patient’s clinical history and radiographic findings were unremarkable. First, the UTE-MRI images were randomly assigned. A reader was required to record and label every visible lung nodule. Moreover, the observer used a 5-point rating scale for subjective image quality assessment of overall UTE: MR images (5 = perfect, 4 = good, 3 = diagnostic, 2 = bad, 1 = unacceptable), vascular conditions (5 = perfect, subsegmental blood vessels, 4 = good, subsegmental blood vessels, 3 = diagnostic, segmental blood vessels, 2 = bad, lobe vessels, more artifacts in each vessel, 1 = unacceptable, unable to distinguish lobar vessels), and bronchial conditions (5 = perfect, subsegmental bronchus visible, 4 = good, subsegmental bronchus visible, 3 = acceptable, segmental bronchus visible, 2 = poor, lobar bronchus visible, 1 = unacceptable, unable to distinguish lobar bronchus). To ensure the accuracy of the presence of pulmonary nodules, observers were required to reevaluate the CT images. The observer was unaware of the image features, previous reports, and results of the UTE-MRI image analysis. Third, the observers recorded and labeled all pulmonary nodules. Furthermore, the short diameters of all the detected nodules in the lungs were also recorded. If the same pulmonary nodule was marked and measured by all 3 observers, the average diameter of all measured values was used for further analysis. Otherwise, CT analysis by a 12-year observer was used as the gold standard along with MRI.

### 2.5. Statistical analysis

SPSS version 27 (IBM, Armonk, NY) was used for all the statistical analyses. Continuous variables were expressed as mean ± standard deviation. The Kolmogorov–Smirnov test was used to assess the normality of the data distribution. Normally distributed data were analyzed using the *t* test. The Wilcoxon rank-sum test was used to assess unequal variances. Statistical significance was set at *P* < .05. The accuracy of each observer in detecting pulmonary nodules on UTE-MRI images compared to CT images was calculated if *T* < 1. Fisher exact test was used, and statistical significance was set at *P* < .05. Kendall test was used to measure the consistency of lung nodule detection and image quality evaluation by the 3 readers. A Kender Harmonious Coefficient (W^a^) < 0.2 indicates a poor degree of consistency; a value between 0.2 and 0.4 indicates a general degree of consistency. A value between 0.4 and 0.6 indicates a moderate degree of consistency. A value between 0.6 and 0.8 indicates a strong degree of consistency; a value between 0.8 and 1.0 indicates a high degree of consistency.

## 3. Results

**Figure 2.**
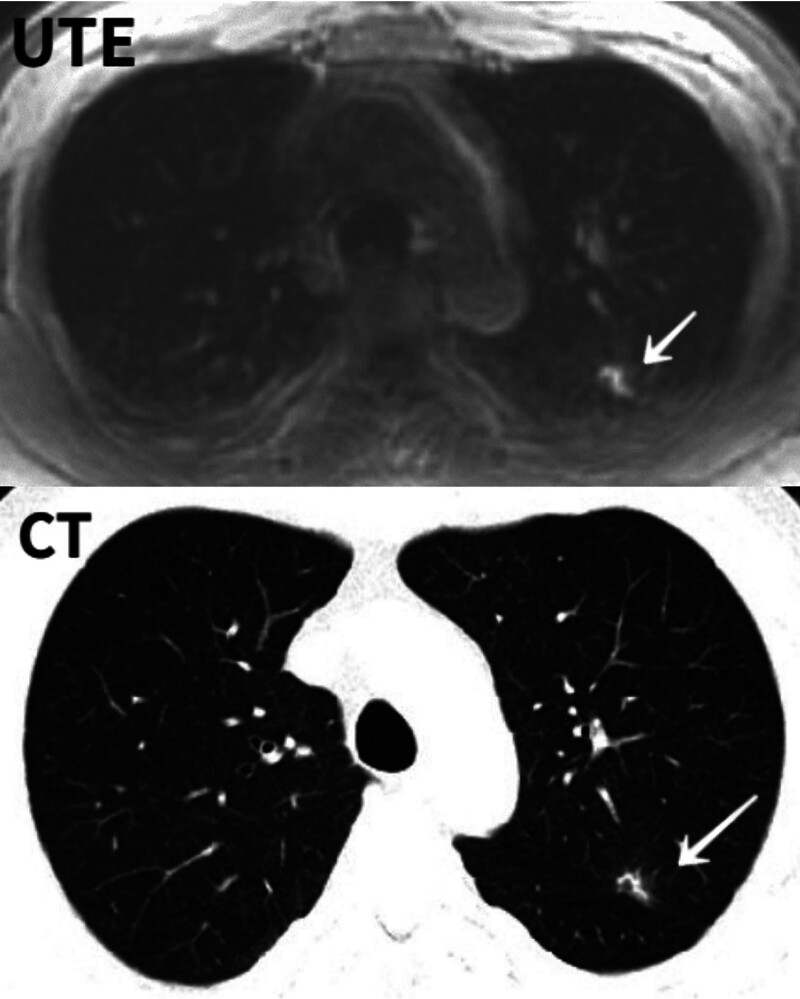
Clearly shows the shape of the ground glass nodules.

**Figure 3.**
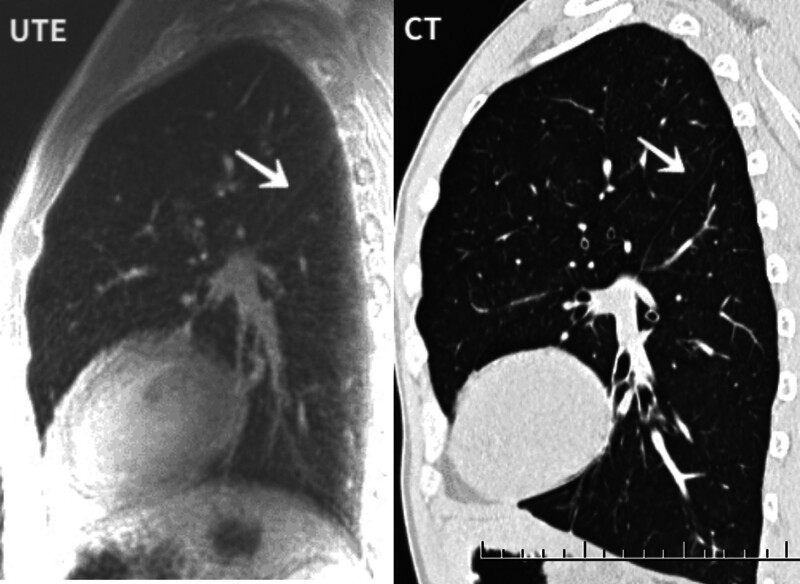
Sagittal view. CT and UTE images were of good overall quality, and the interlobar fissure was clearly visible. UTE = ultrashort echo time.

**Figure 4.**
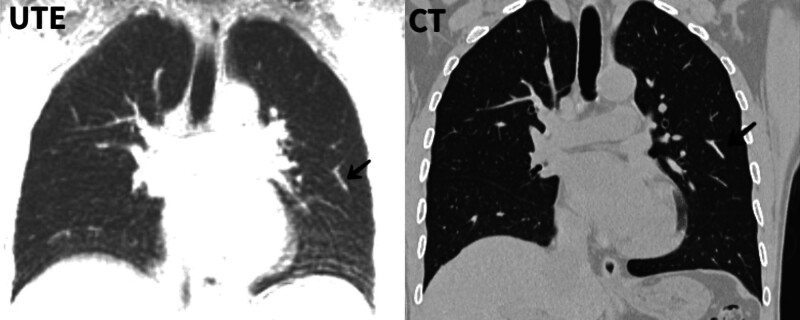
Small vessels clearly showing subsegmental vessels.

**Figure 5.**
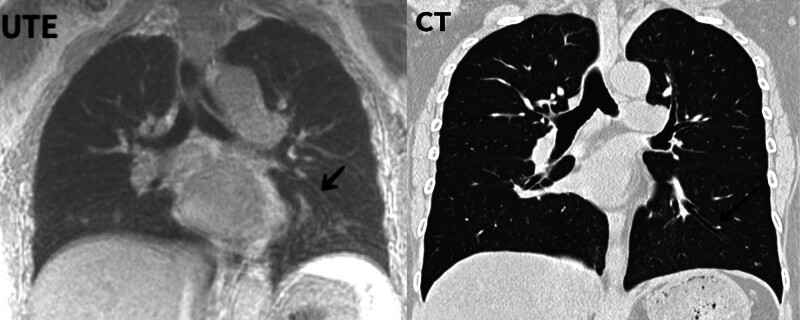
Subsegmental bronchus with a lumen of approximately 2 mm.

The data of 51 patients were collected, 5 of whom did not undergo MRI, resulting in a total of 46 patients (27 men, 19 women; mean age 54.5 ± 12.5 years) who underwent CT and MRI scans after enrollment in the study. Fourteen patients presented with primary lung cancer (non-small cell lung cancer, n = 12; small cell lung cancer, n = 2). Three patients had inflammatory nodules, and no pathological results were obtained for the other patients. The mean CT dose index volume was 7.97 ± 1.50 mGy, and the mean dose-length product was 344 ± 40.4 mGy-cm. On routine CT images, lung nodules were present in 36 patients (78.2%) but not in 10 patients (21.7%). A total of 48 pulmonary nodules were also identified. The average nodule diameter was 8.69 ± 3.13 mm, ranging from 4.5 to 15.9 mm, 3 of which were ground glass nodules (Fig. 2). UTE-MRI revealed 48 lung nodules in 36 patients (Table 1). Compared to CT, the sensitivity of all MRI readers for detecting lung lesions was 95.8%, and the 3-observer agreement was nearly perfect (*P* < .001, Kendall W^a^ = 0.913), as shown in Table 1. Compared with the reference standard CT, 2 false-positive lesions were recorded by 1 observer, and 1 false-positive lesion was recorded by 2 observers, with a mean diameter of 5.6 ± 0.5 mm and a range of 5.1 to 6.1 mm. The mean short diameter measured using UTE-MRI was approximately 8.70 mm, which was smaller than that measured using CT (8.98 mm), and there was a significant difference between them (*P* < .001, *z*-value = ‐4.365). The overall image quality score of the observers was high, ranging from good to perfect (Fig. 3), and the consistency of the UTE-MRI subjective image quality was excellent (Kendall W^a^ = 0.877, *P* < .001). The subsegment of the bronchus was displayed for visualization of the trachea, as shown in Figure 4, and the wall of the tube was clearly displayed. Subsegmented blood vessels were also observed (Fig. 5), with clear walls and uniform signals (Table 2).

**Table 1:** Lung nodules detected via UTE by 3 observers (with CT as the gold standard).

	UTE detection (case)	Mean diameter measured in UTE (mm)	CT detection (case)	Mean diameter measured in CT (mm)	False positive in UTE (case)	Consistency of 3 observers in UTE images	
						*P* value*	Kendall W^a^
Observer 1	48	8.70 + 3.07	48	8.98 + 3.02	2	*P* < .001	0.913
Observer 2	47	8.71 + 3.05	48	8.97 + 3.08	1		
Observer 3	47	8.66 + 3.13	48	8.96 + 3.15	1		

UTE = ultrashort echo time, mm = millimeter.

* *P* < .05, indicating statistical significance.

**Table 2:** Average scores and consistency of the 3 observers in UTE images.

	Average score	Kendall W*	*P* value†
Overall image quality	4.33 ± 0.51	0.877	*P* < .001
Tracheal display	4.03 ± 0.54	0.804	*P* < .001
Blood vessels display	4.34 ± 0.56	0.823	*P* < .001

UTE = ultrashort echo time, mm = millimeter.

* Kendall coefficient of concordance.

† *P* < .05, indicating statistical significance.

## 4. Discussion

With improvements in CT utilization and resolution, an increasing number of lung nodules with uncertain malignant tumors have been detected.^[11]^ However, the differences between benign and malignant CT imaging features as well as the use of solid components to indicate malignant tumors and their prognosis are still controversial.^[12]^ Positron emission tomography containing F-18 fluorodeoxyglucose has not been shown to be valuable for the assessment of pulmonary nodules, especially ground-glass duration.^[13]^ There are significant radiation risks, and a possible need for further invasive procedures and postoperative complications. In recent years, 3.0 T MRI technology has gradually matured and become widely used, and magnetic susceptibility, contrast, and chemical shift resolution have improved, including the emergence of faster image acquisition technology, which can more clearly show normal anatomic structures and better identify diseased tissues.^[14]^ The UTE sequence developed in recent years is primarily used to acquire the short T2 component, and its ultrashort TE value is used to rapidly acquire the MR signal of the short T2 component before rapidly decaying to zero. Because lung tissue contains a large amount of gas, the T2 time is very short, and signal acquisition is limited. The UTE sequence solves this problem.

UTE-MRI is highly consistent with CT in displaying the characteristics of lung diseases (such as ground-glass opacity, consolidation, nodules, and fibrosis), and its image quality is similar to that of CT, which is considered of great value in evaluating various lung parenchymal diseases.^[10]^ Additionally, a study by Delacoste et al showed that the image quality of UTE-MRI for quantifying the volume of pulmonary nodules was similar to that of CT.^[15]^

This study revealed that UTE had a high detection ability for pulmonary nodules, which was close to that of CT, with a detection rate of 95.8%, and it could also detect some ground-glass nodules. However, although UTE-MRI sequences allow rapid acquisition time and diagnostic image quality, our results suggest that high-resolution CT is still necessary for the accurate detection of nodules <5 mm in diameter, as well as ground-glass nodules, such as those detected simultaneously by all observers in some cases. A 5 mm lesion and nodule were missed on magnetic resonance imaging. The main reasons for this are as follows: First, the K-space center in MRI image acquisition is low-frequency information, which mainly determines the image comparison; second, the K-space is surrounded by high-frequency information, which determines the edges and details of the image. For lung MRI, the use of a radially or spirally sampled K-space to compensate for respiratory movements and a very short T2* result in undersampling of the peripheral K-space, which results in blurred images.^[16]^ Third, some nodules were smaller and had a lower density, indicating a ground-glass composition. Fortunately, at least 95% of lung nodules are benign, most commonly granulomas or intrapulmonary lymph nodes. Smaller nodules are more likely to be benign, and the histological features may not be clearly defined in most cases.^[17]^ Furthermore, according to recent guidelines,^[18–20]^ nodules < 5 mm are generally not included in screening programs. We also detected several false-positive nodules on UTE-MRI, mainly because our observers misinterpreted blood vessels as pulmonary nodules. Renz et al conducted a study on children and adolescents with malignant tumors and reported that the detection rate of nodules within 1 to 4 mm was 68.3% (43/63). Although patients underwent contrast-enhanced magnetic resonance imaging, this study also provides a basis for expanding the detection range of pulmonary nodule UTE in the future.

**Figure 6.**
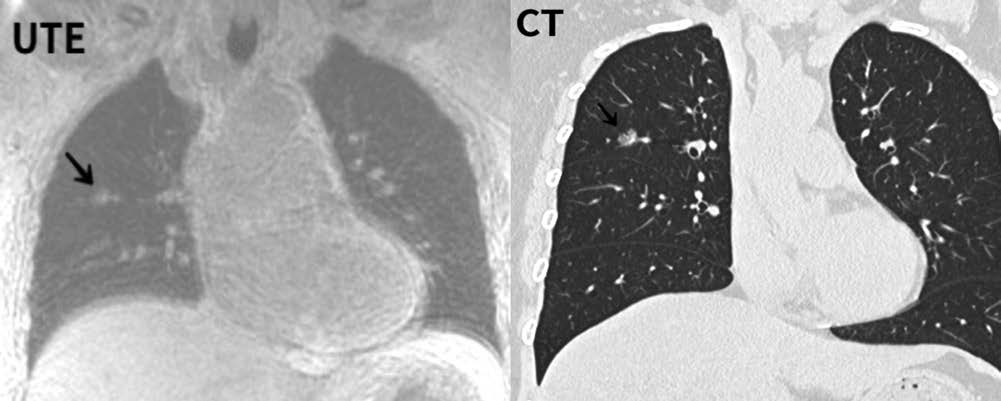
Subsolid nodules. Fewer ground glass opacities were observed on the UTE-MRI images than on the CT images. UTE-MRI = ultrashort echo time MRI.

The results of this study revealed that the average diameter of lung nodules measured using UTE images was smaller than that measured using CT images, and there was a large difference in the measurement results. The main reason may be that at the nodule margins, UTE-MRI is weaker than CT images; some nodules are central solids with ground glass at the edges, and the ground glass parts at the edges are less dense and not clearly displayed (Fig. 6).

Subjective image quality was assessed by 3 observers. We found that the average UTE-MRI score was diagnostic to perfect (3–5 points), mainly good, and weaker than that of CT images. This may be because observers are more acquainted with the grayscale contrast of CT images and the imaging features of lung nodules than with MRI. CT is superior to MRI because of its higher temporal and spatial resolution. Despite this, the study revealed only minor differences in overall image quality and detection performance for nodules larger than 5 mm.

Subsegmental bronchi and blood vessels were observed on UTE-MR images. Although the overall score of the trachea is lower than that of the blood vessels, this is mainly because the trachea contains more air and the interference is more severe. However, it has extensive application value for most lung conditions such as pulmonary edema, bronchitis, and other diseases. Functional pulmonary MR has great potential for evaluating pulmonary function in the future.

Furthermore, interobserver evaluations of UTE-MRI and CT image compactness were nearly perfect, indicating that the image quality of the 2 modes was objectively good and diagnostic, regardless of observer experience.

Finally, the optimal magnetic field strength for MR was determined. Although researchers such as Chassanionon et al^[21]^ compared UTE images of the lungs at 1.5 T and 3 T, the results showed that the image quality at 1.5 T was greater than that at 3 T. However, owing to the need for a higher spatial resolution to improve the precision and accuracy of nodule detection, an increasing number of studies are being conducted at 3 T.

Therefore, UTE-MRI may be used in the future as a partial replacement for CT, especially in infants who are not eligible for radiation, or in patients who require MRI but additional lung CT to follow-up pulmonary nodules or PET-MRI. Patients with known pulmonary nodules > 5 mm may be followed-up with MRI or alternatively with CT to reduce the radiation dose.

Our study had several limitations. First, we did not compare MRI sequences with other MRI sequences previously reported to be highly sensitive and specific for detecting lung lesions.^[4]^ We used CT as the gold standard, and this study focused on the UTE sequence assessment. Second, the number of patients was relatively small and no analysis was conducted on the subtypes of pulmonary nodules (e.g., solid and subsolid), although our study included some ground-glass nodules. Third, the multiple lung nodules showed no pathological findings. Our study mainly assessed the image quality and nodule detection rate, which were not related to pathological results. In the future, more cases will be included, and further studies will be conducted to compare UTE-MRI with pathology to establish whether UTE-MRI can substitute repetitive pulmonary CT in higher-risk patients.

In conclusion, compared to CT, UTE-MRI can detect pulmonary nodules with a high detection rate, relatively good image quality, and strong consistency between observers. Therefore, UTE-MRI will become a stable imaging method for the detection or follow-up of pulmonary nodules, and for the diagnosis of pneumonia when ionizing radiation is reduced.

## Author contributions

**Conceptualization:** Liuhong Zhu.

**Data curation:** Chong Lin.

**Writing – original draft:** Xiaoxia Li.

**Writing – review & editing:** Funan Wang.
